# Québec Wildfires and Respiratory Emergency Department Visits in Charlottesville, Virginia

**DOI:** 10.1007/s10393-026-01783-9

**Published:** 2026-03-18

**Authors:** Luis M. Sanabria Segura, Wendy M. Novicoff, Kyle B. Enfield, Sara Kakatkar, Robert E. Davis

**Affiliations:** 1 School of Medicine, University of Virginia, Charlottesville, VA 22903 USA; 2 Departments of Public Health Sciences and Orthopedic Surgery, University of Virginia, Charlottesville, VA 22908 USA; 3 Department of Environmental Sciences, University of Virginia, Charlottesville, VA 22904-4123 USA

**Keywords:** wildfires, air quality, emergency department visits, respiratory, Charlottesville, Virginia, Québec, Canada

## Abstract

The decline in air quality from wildfires is known to have detrimental health impacts locally, but less is known about the effects far from the source. To address this gap, we analyzed visits to the University of Virginia Emergency Department from 2017 to 2023 by examining the impact of elevated PM_2.5_ levels on respiratory visits. For this analysis, high exposure days were defined as those with PM_2.5_ levels 2 or more standard deviations above the 2017–2023 average, specifically during the summer of 2023 when the Québec wildfires plume was advected over central Virginia. The results showed higher odds ratios on high PM_2.5_ days compared to dates with normal exposure (1.190 [1.026,1.380]). This result was observed in males (1.388, [1.122,1.716]) and in white individuals (1.220 [1.009, 1.475]). Additionally, a comparison of mean ED visit departures (detrended and deseasoned) revealed that same day respiratory visits were significantly elevated on high exposure days (mean departure difference =  + 1.886, *p* = 0.027). These findings indicate that wildfires can have measurable health impacts in areas far from their origin.

## Introduction and Purpose

**Figure 1:**
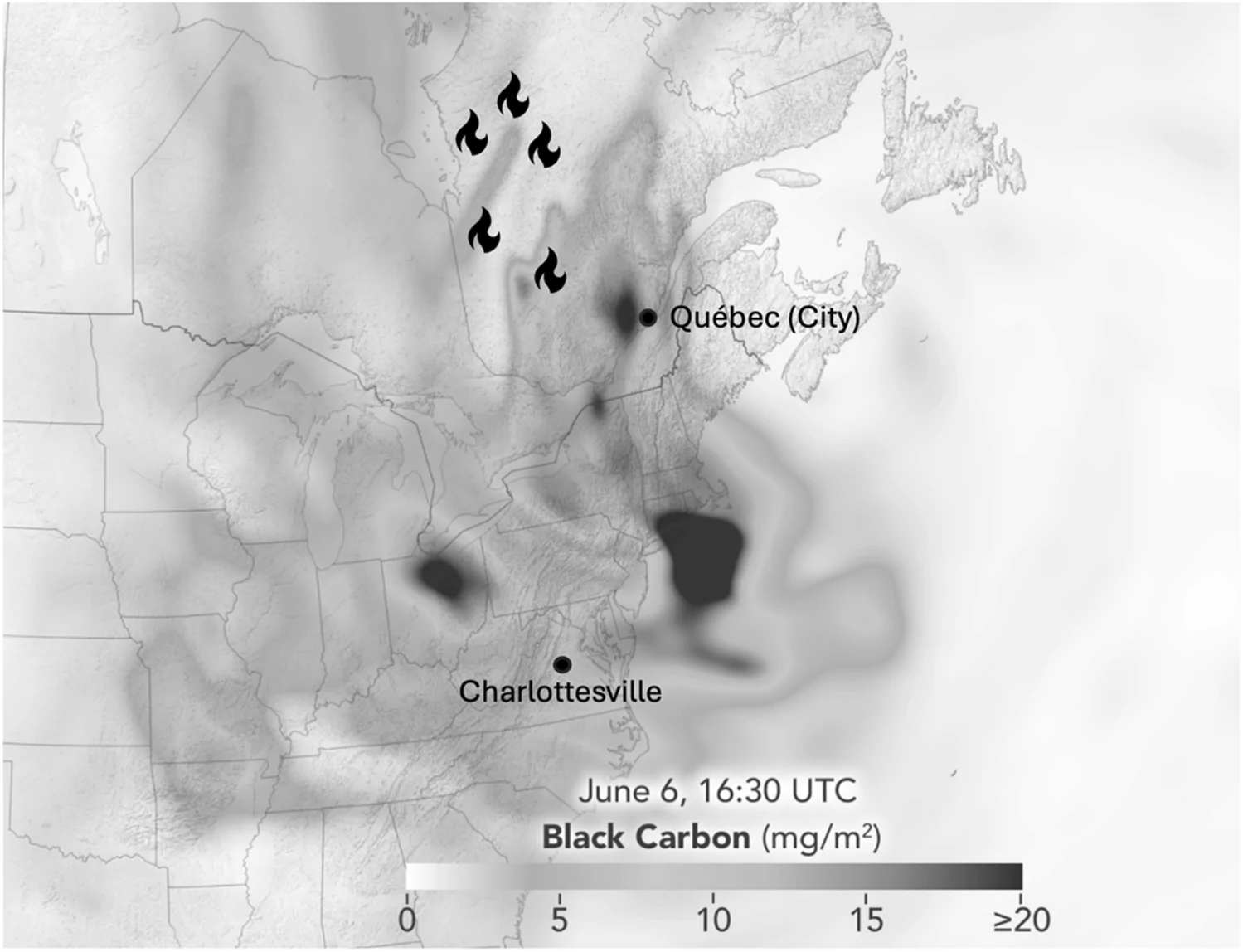
GEOS-FP satellite image on June 6, 2023, showing black carbon concentrations over the northeastern U.S. and southeastern Canada (Dauphin, 2023). A general indication of some of the major fire sources is indicated by the fire symbol. A time series animation can be accessed at: https://earthobservatory.nasa.gov/images/151442/hazardous-air-chokes-northeastern-states. Accessed on November 13, 2025.

The intensity and frequency of wildfires and the duration of the wildfire season are expected to increase in response to anthropogenic climate change (Flannigan et al., 2013; Masson-Delmotte, 2019; Sun et al., 2019; Zeng et al., 2019). Between March and October 2023, over 6500 fires in Québec burned over 4 million hectares, the highest on record for Canada (Center for International Forestry Research, 2024). The environmental impact of these events is not always limited to the region immediately adjacent to the origin of the fires. In June and July 2023, smoky conditions and dangerous air quality levels were evident throughout most of the eastern United States (Figure 1), reaching as far south as Florida and as far east as Europe (Copernicus Atmospheric Monitoring Service, 2024). Air pollution levels in several major American cities were among the worst in the world.

Most prior research has focused on the impact of respiratory illnesses in the general vicinity of wildfires (Liu et al., 2017; Chen et al., 2023a; Doubleday et al., 2023; Douglas-Vail et al., 2023; Heft-Neal et al., 2023). There is also evidence for further impact of wildfires on mortality and morbidity such as increases in respiratory, cardiovascular, and all-cause mortality, all-cause cardiovascular emergency department visits, cerebrovascular accidents, physician visits, hospital admissions, and diminished lung function in individuals with no history of reactive airway disease, among others (Wettstein et al., 2018; Reid et al., 2016; Elser et al., 2023; McArdle, 2023). Lagged (1–2 days) increases in respiratory admissions affecting patients with pre-existing conditions have also been shown (Skinner et al., 2022). Furthermore, wildfires have been linked to increased rates of a host of psychiatric conditions such as post-traumatic stress disorder, major depressive disorder, and anxiety (To et al., 2021) in both children and adults. Studies have shown similar impacts in other countries affected by wildfires, such as Brazil and Australia (Requia et al., 2021; Ye et al., 2022; Ademi et al., 2023; Barbosa et al., 2023).

The One Health approach recognizes the interconnectedness between human health and the surrounding environment (Auplish et al., 2024). In that context, few studies have explored how a remote environmental event can influence health over a large geographic expanse. Herein, we examine whether the reduction in air quality resulting from wildfires impacted health in a region significantly distant from the origin of the fires. Specifically, we study the influence of high PM_2.5_ concentrations during the period of the Québec wildfires in the summer of 2023 on emergency department visits for respiratory conditions at the University of Virginia Hospital in Charlottesville, Virginia, a location over 1100 km from Québec city (Fig. 1).

## Data and Methods

Individual patient-level Emergency Department (ED) visits to the University of Virginia Medical Center in Charlottesville, Virginia, were obtained from January 1, 2017, through December 31, 2023. ED visits for patients with respiratory diseases as a primary diagnosis were subsetted from the master file and totaled daily. As our purpose is to examine wildfire impacts, we excluded pneumonia and influenza (International Classification of Diseases, Version 10, codes J10–J18) as well as COVID-19 (Table 1). Our study included all ED patients without consideration of their place of residence.

**Table 1:** International Classification of Diseases, Version 10 (ICD-10) codes and the associated diagnoses utilized in this study.

ICD-10 Code Categories	Diagnoses
J00 – J06	Acute upper respiratory infections
J20 – J22	Other acute lower respiratory infections
J30 – J39	Other diseases of the upper respiratory tract
J40 – J47	Chronic lower respiratory diseases
J60 – J70	Lung diseases due to external agents
J80 – J84	Other respiratory diseases principally affecting the interstitium
J94	Other pleural conditions
J96 – J99	Other diseases of the respiratory system
R05 – R07	Cough, abnormalities of breathing, and pain in throat/chest

**Figure 2:**
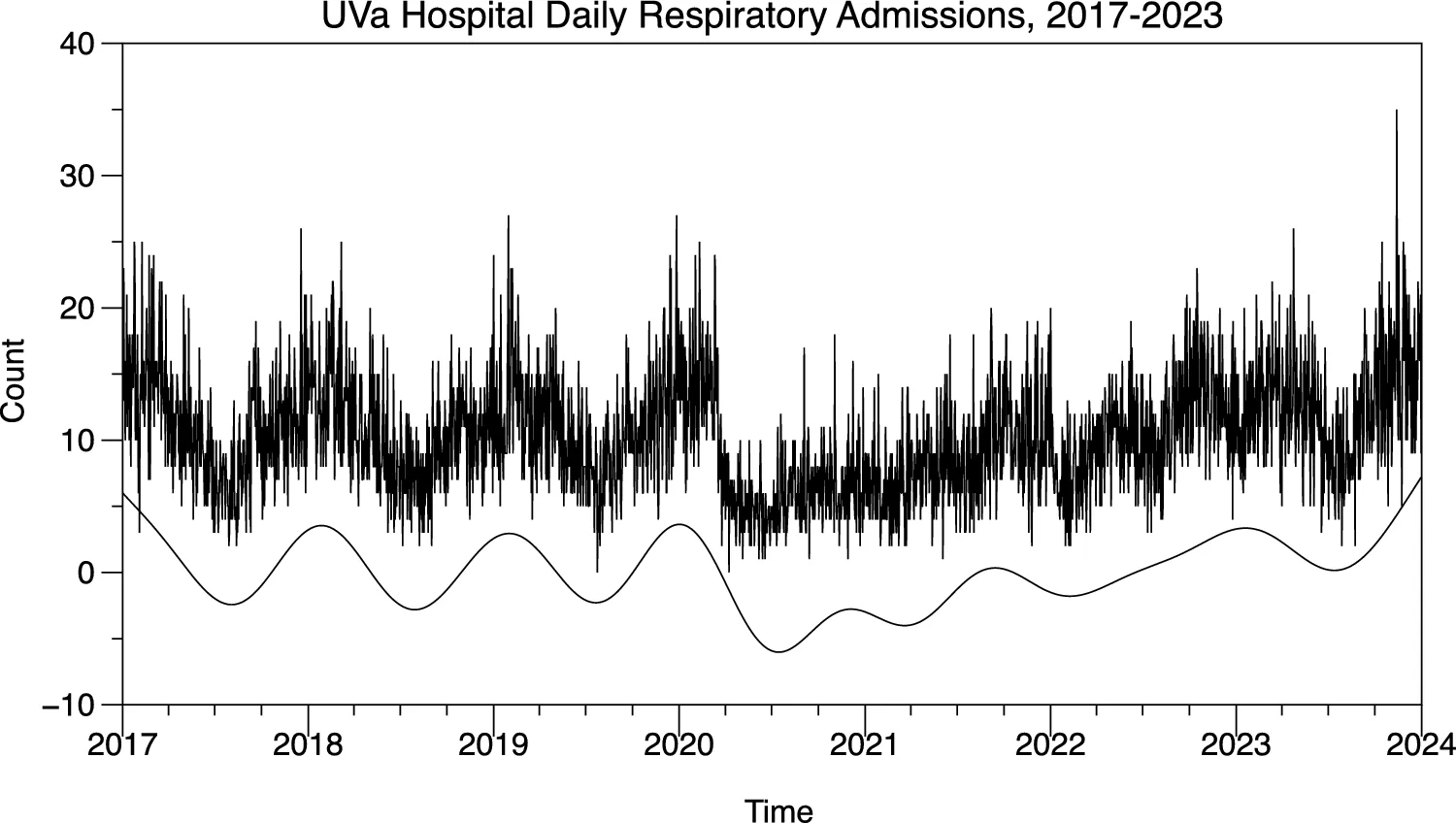
University of Virginia Emergency Department Respiratory Visits (2017–2023). The dataset excludes visits for COVID-19, influenza, and pneumonia. The generalized additive model fit with 3 degrees of freedom per year is shown by the smooth line, which is plotted below the ED visit time series to improve readability.

Data were organized into daily visit counts by gender, age (0–17, 18–64, and ≥ 65 years), and race/ethnicity. (Because of sample size limitations, we could not analyze African American, Native American, or Asian subgroups.) The time series exhibits clear seasonality (Fig. 2) with higher ED visits in winter. The time series is characterized by an obvious discontinuity in spring, 2020, when the onset of the COVID-19 pandemic suppressed less acute ED visitation. The time series returned to a more typical seasonal pattern by 2023. Herein, we focused only on summer ED visits, defined as June 1 through July 31 (the Québec wildfires peaked from June through early July 2023).

As of the year 2022, 46,289 people were living in the city of Charlottesville (65.30% white, 17.00% Black or African American, 6.99% Asian, and 5.84% Hispanic). The demographic distribution of our dataset was relatively similar, with 67.61% of patients identifying as white, 23.2% as African American, and 7.55% as Hispanic. UVA Health is an integrated health system consisting of a 650-bed academic medical center in Charlottesville, Virginia, three community hospitals, more than 200 outpatient clinics, and both a medical school and a nursing school. Although the hospital is in an urban area, it acts as the only safety-net hospital in the western two-thirds of the state of Virginia and is a regional referral center for approximately 87 counties in Virginia and West Virginia. As such, it serves the population of a large geographic area. The UVA ED is a 70-bed, Level 1 trauma center that treats adults and children, with a separate rapid medical evaluation area for minor emergencies and a dedicated section for behavioral and mental health emergencies. UVA Hospital’s Emergency Department (ED) registered more than 72,000 visits in 2023. In 2023, the entire ED population was 52.0% female, 64.2% White/Caucasian, and 10.8% Hispanic, with a mean age of 42.0 years (standard deviation = 24.6, range 0 to 88 years). (Note that a substantial percentage of patients choose not to identify their race and/or ethnicity, and this accounts for much of the missing demographic data.)

**Figure 3:**
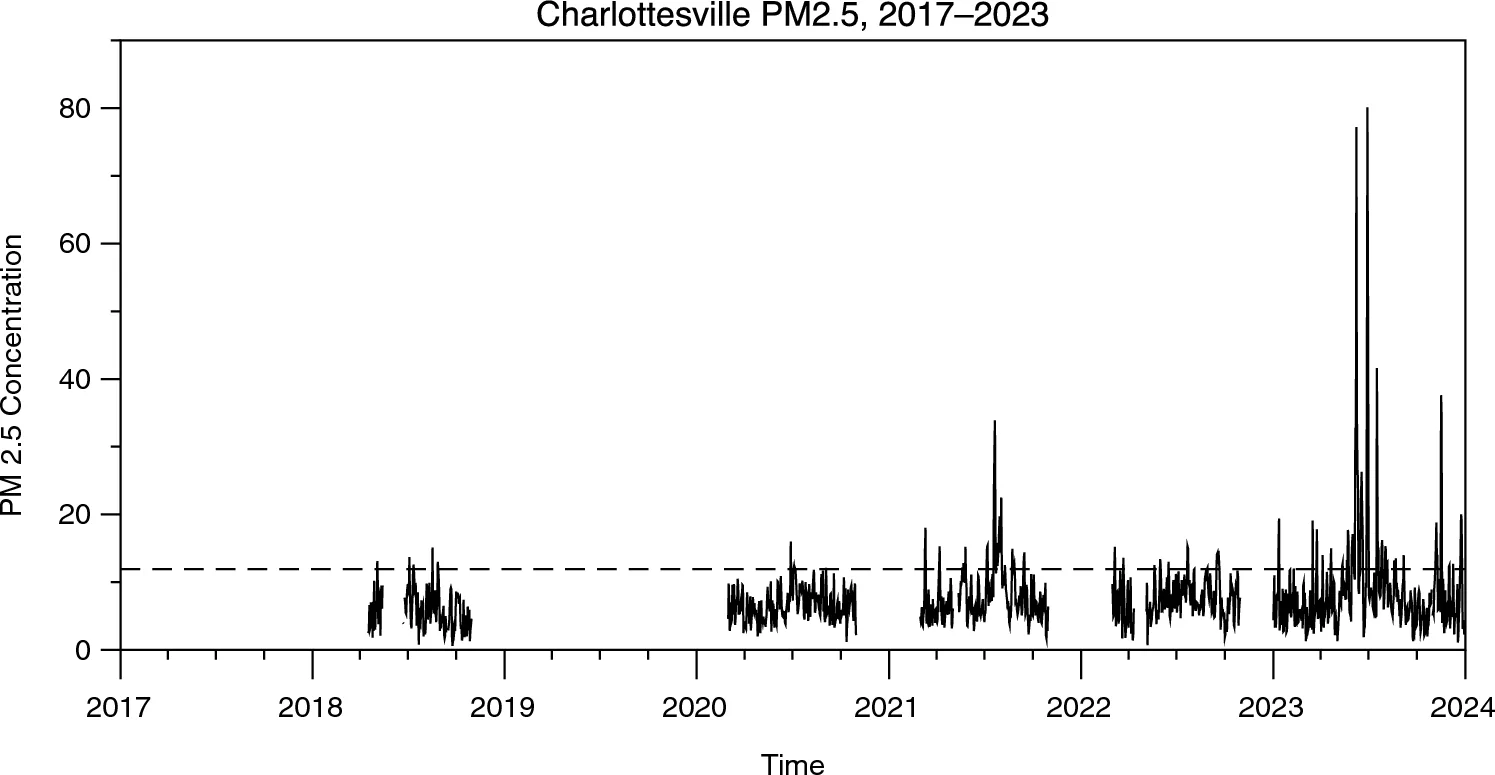
Daily mean PM_2.5_ Concentrations in Charlottesville, Virginia. The threshold PM_2.5_ value (11.92 µg m^–3^) is indicated by the dashed horizontal line.

We obtained PM_2.5_ (particulate matter less than 2.5 µm in diameter) data for Charlottesville, Virginia from the EPA (US Environmental Protection Agency, 2024) from 2017 through 2023. Observations were taken at a single site (Albemarle High School) and were recorded either every 3 days (using an R&P Model 2025 PM-2.5 Sequential Air Sampler) or daily (via a Teledyne T640), depending on the time period. All observations were reported as daily mean concentrations (in micrograms per cubic meter). There were notable gaps in the available data for 2017 and 2019, so only days with reported PM_2.5_ measurements were used for our analysis. The daily PM_2.5_ time series shows uncharacteristically high concentrations in June and July 2023 (Fig. 3). We used a threshold approach to identify high PM_2.5_ days (Chen et al., 2023b) as those days with concentrations at least two standard deviations greater than the pre-COVID (2017–2019) mean (mean = 6.14; threshold = 11.92). We subsequently refer to days above the threshold as "case" or "high exposure" days and all others as "control" or "normal exposure" days.

Because of the extent of missing data in the PM_2.5_ time series, a regression-based approach (such as a time-stratified case-crossover) was untenable. Instead, to determine if ED visits exceeded expectations during the high smoke events, we compared the mean ED visit totals on days with high exposure (cases) vs. days with normal exposure (controls). To remove potential confounding related to the trend, seasonality (Fig. 2), and day-of-week effect in the ED data (one-way analyses of variance for day-of-week used a Type I error rate of p ≤ 0.05), we ran a generalized additive model by fitting each year with penalized regression splines with 3 degrees of freedom per year. The data were assumed to have a Gaussian distribution, and the degrees of freedom were selected after comparing various options to account for the trend without overfitting. Thus, we adjusted for anomalies in the time series, such as the COVID-19 epidemic and its aftermath. Analyses were run using the "mgcv" package in R Studio (Version 2024.04.2 + 764; Wood, 2000). The daily departures from the model’s predictions, or expected mean daily ED visits, were used as the new response variable. Dates were selected from June 1–July 31, 2023, the period of peak wildfire effects. To examine potential lags, ED visit departures were reviewed on the day when the PM_2.5_ threshold of 11.92 ug/m^3^ was exceeded and the subsequent 2 days. We selected a 2-day lag for consistency with prior research on the impact of acute wildfire exposure on morbidity (Alman et al., 2016; Liu et al., 2017; Chen et al., 2023b, a). Group means were compared using one-tailed t-tests with a Type I error rate of 0.05.

In addition, we calculated odds ratios (ORs) using the raw visit counts. Exposure was defined as days with PM_2.5_ concentrations at or above the threshold, whereas non-exposure dates were those below the threshold. Diseased subjects presented to the ED for a respiratory disease and non-diseased subjects presented for all other reasons. In situations where the entire population is potentially exposed, it is impossible to identify pure "controls" in a traditional sense. We therefore used all non-respiratory ED visit totals for this purpose. If there are possible non-respiratory comorbidities associated with smoke exposure, these would tend to lower the OR estimates. Thus, we believe this is a statistically conservative approach for estimating the OR. Again, all high exposure days were selected from June 1–July 31, 2023. Normal exposure days were date-matched from June and July of 2018–2022 (summer, 2017 was not used because of missing data). Only dates with recorded PM_2.5_ data were included. As a robustness check, we also calculated ORs using a similar method but obtained normal exposure days from summer of 2023 only. The format of the OR calculations is presented in Table 2.

**Table 2:** Set-up for odds ratio calculations a) using date-matched normal exposure days from the summers of 2018–2022 and b) using normal exposure days in the summer of 2023.

a)	Respiratory ED Count	Non-respiratory ED Count
High exposure days (PM_2.5_ ≥ threshold)	June/July 2023	June/July 2023
Normal exposure days (PM_2.5_ < threshold)	June/July 2018–2022 (matched by day)	June/July 2018–2022 (matched by day)

b)	Respiratory ED Count	Non-respiratory ED Count
High exposure days (PM_2.5_ ≥ threshold)	June/July 2023	June/July 2023
Normal exposure days (PM_2.5_ < threshold)	June/July 2023	June/July 2023

In both approaches, an underlying assumption is that the entire population was exposed to high particulate levels on days that exceed the threshold, as well as to their ambient environment on all other days. The Québec smoke plume was a persistent feature in the summer of 2023. Although the PM_2.5_ concentrations varied over time and space in response to changes in atmospheric wind and stability patterns, the hazy conditions associated with northerly winds were uncharacteristic for this area (Fig. 1).

## Results

In our examination of mean differences in ED visit departures, 26 case dates (high exposure) and 23 control dates (normal exposure) were identified for the same day analysis, 28 controls were obtained for the 1-day lag, and 35 controls were selected for the 2-day lag.

**Figure 4:**
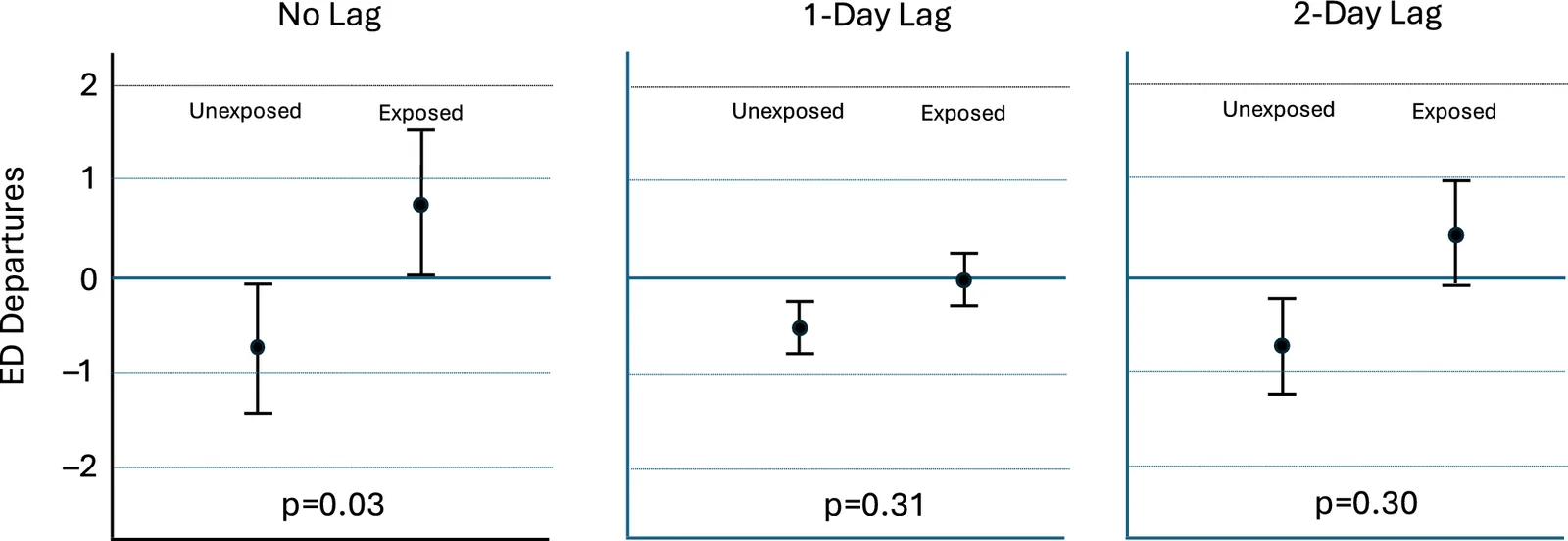
Response of ED Visit Departures to High and Normal PM_2.5_ Concentrations. Associated *p*-values for one-tailed t-tests with alpha = 0.05 are included. Error bars are 1 standard error of the mean.

We found an increase in same day ED visits on days with elevated PM_2.5_ concentrations coincident with the Québec wildfires (Fig. 4). No increase was evident between mean departures at 1- or 2-day lags.

For the OR calculations, we identified 26 case dates during June–July of 2023 and 75 control dates from previous summer periods. We found an increase in the odds of presenting for a respiratory disease in the exposed group compared to the unexposed (Table 3). This effect was present in the entire population (OR = 1.19 [1.03, 1.38]) as well as for men (1.39 [1.12, 1.72]) and white patients (1.22 [1.01, 1.48]). No effect was found for women, Hispanics, or any of the age groups examined.

**Table 3:** Odds ratios (OR) with associated 95% confidence intervals (95% CI)

Population	OR	95% CI	High Exposure	Normal Exposure
All	1.190	1.026–1.380	4,613	11,349
Men	1.388	1.122–1.716	2,234	5,421
Women	1.027	0.834–1.266	2,376	5,928
Age ≤ 17 years	1.147	0.880–1.496	752	1,698
Age 18–64 years	1.075	0.872–1.326	2,652	6,681
Age ≥ 65 years	1.180	0.881–1.582	1,209	2,970
White	1.220	1.009–1.475	3,058	7,766
Hispanic	1.254	0.778–2.021	441	756
Non-White	1.125	0.886 –1.427	1,555	3,583

Normal exposure days were obtained from summers 2018–2022. The number of visits is listed for both the high and normal exposure groups. Bold font highlights populations with OR CIs that do not include 1.0.

Focusing the analysis only on the summer of 2023, we identified 26 case dates and 35 control dates. We see a similar positive effect on the entire population (OR = 1.23 [1.04, 1.46]) and men (1.32 [1.04, 1.67]) but found no relationship for women (Table 4). No significant relationships were identified for any of the race/ethnicity or age-based subgroups.

**Table 4:** Odds ratios (OR) with associated 95% confidence intervals (95% CI)

Population	OR	95% CI	High Exposure	Normal Exposure
All	1.234	1.044–1.459	4,613	6,328
Men	1.319	1.041–1.672	2,234	3,066
Women	1.155	0.911–1.464	2,376	3,259
Age ≤ 17 years	1.182	0.881–1.588	752	1,013
Age 18–64 years	1.182	0.932–1.489	2,652	3,752
Age ≥ 65 years	1.096	0.790–1.520	1,209	1,563
White	1.219	0.982–1.512	3,058	4,068
Hispanic	1.600	0.954–2.684	441	665
Non-White	1.275	0.977–1.663	1,555	2,260

Normal exposure days were obtained from the summer of 2023 only. The number of visits is listed for both the high and normal exposure groups. Bold font highlights populations with OR CIs that do not include 1.0.

## Discussion

We found an increase in ED visits on days with elevated PM_2.5_ concentrations associated with the extensive wildfires in Québec during June and July of 2023. The wildfire impact was acute and did not extend to 1 or 2 days after the high PM_2.5_ event, a result that is consistent with the literature examining more localized fire-related ED impacts (Alman et al., 2016; Liu et al., 2017; Chen et al., 2023b, a). Furthermore, results showed an increased odds of presenting to the ED for a respiratory complaint on high- vs. normal-exposure days, consistent with other studies (Chen et al., 2023a; Douglas-Vail et al., 2023; Center for International Forestry Research, 2024; Copernicus Atmospheric Monitoring Service, 2024). Interestingly, this effect was present for men in both OR calculations and for white patients in the date-matched approach. The ORs for women, however, were not significant. Literature on differing responses by gender is inconsistent and varies by the nature of the chief complaint. Although some studies suggest women comprise the majority of ED and hospital admissions for asthmatic syndromes (Baibergenova et al. 2006), others have shown that men account for 60.1% of total ED admissions (Anson et al., 1991). When focusing on a more extensive range of respiratory conditions, rates of admission to the hospital from the ED are lower for women (7.2%) than men (19.2%) (Satia et al., 2021).

None of the three age groups examined saw a significantly elevated OR. Chen et al. (2023a) found no significant increase in the asthma-related ED visit incidence rate ratio in New York City for the 0–4 years and ≥ 65 years age groups. As most of these studies have been carried out in individual hospitals or cities, this opens the possibility for small sample sizes underpowering statistical analyses. This likely contributed to the lack of significant effects in some of our subgroup analyses.

This study demonstrates that environmental impacts can extend far beyond the local source. Charlottesville, Virginia, approximately 1100 km southwest of Québec City, was impacted by uncharacteristically high PM_2.5_ levels on a few days in June 2023 (Fig. 3) and residual elevated concentrations on other days during that summer. The odds of presenting to the ED for respiratory conditions were 19–23% higher on days with poor air quality. In other words, the results show that smoke from a distant fire (Sanford-Townsend Band, 1977) impacted health locally. As wildfires are occurring with increased frequency and severity, it is reasonable to suggest that such effects will continue to grow in the coming years. It would be interesting for future research to study any compounding effects the wildfire-induced worsening of air quality may have on health in larger cities with already high PM_2.5_ reference values.

The study’s limitations are essential to discuss to better contextualize some of the results. The data were obtained from one health system in a small city, thus limiting the daily sample size and potential statistical robustness. This is amplified by the number of days with high PM_2.5_ and is a critical point when considering our subgroup analysis. In terms of our power analysis, for 20% relative precision, a 95% confidence interval, an expected prevalence in the “non-exposed” group of 5%, an expected odds ratio of 1.2, and a ratio of presence to absence of about 2, we would need approximately 2100 patients in each group to have enough power to detect statistical significance. Therefore, some of our subgroup analyses are underpowered. Air quality data were gathered for a single site that might not be representative of the greater region, although we believe this error to be comparatively small given the widespread nature of this haze event (Fig. 1). It is impossible to know each individual’s exposure, so we implicitly assume that everyone has been exposed to their ambient environment. We likewise assume that patients who presented to the ED for non-respiratory complaints were unaffected by PM_2.5_ exposure, but this is likely not true for some patients. Several studies have identified an impact of poor air quality on non-respiratory conditions (Liu et al., 2017; Chen et al., 2023a; Doubleday et al., 2023; Douglas-Vail et al., 2023; Heft-Neal et al., 2023). However, the inclusion of these possible air quality-related comorbidities would artificially lower our OR calculations, so our method was conservative with respect to this issue.

Whether the heterogeneity of the hospital is representative of the population at large is a challenging question to answer; however, the results obtained were consistent with those of studies undertaken at different hospitals and cities. For example, numerous studies have found the respiratory ED response to be acute, with a quickly declining impact after 1 or 2 days (Liu et al., 2017; Chen et al., 2023a, b; Doubleday et al., 2023; Douglas-Vail et al., 2023) Although the methods vary, the ED wildfire impact of a several percent increase was generally consistent for other locations, including some studies where the wildfire sources were much closer to the affected population (Liu et al., 2017; Chen et al., 2023a; Doubleday et al., 2023; Douglas-Vail et al., 2023; Heft-Neal, 2023). The available data limited some of the subgroup analyses in recent years, with an observed decrease in the number of patients who chose to self-identify as African American upon admission to the ED. For this reason, we could not include African American, Asian, and Native American categories in our analysis. The use of an administrative database can be challenging as the availability of some demographic information may change over time. We are also aware that socioeconomic factors could influence some of our results, such as availability of transportation to the hospital, paid time off, and insurance coverage, which can impact ED access to specific population groups. However, our dataset and sample size preclude such an analysis.

Previous studies indicate that exposure to wildfire smoke is not distributed equally, with communities of higher Social Vulnerability Index (SVI) having a disproportionately larger number of smoke exposure days (Jung et al., 2023; Modaresi et al., 2023; Vargo et al., 2023; Casey et al., 2024; Schollaert et al., 2024; Dennin et al., 2025). U.S. Census tracts in the highest tertile include communities with limited English proficiency, crowded housing, and lower educational attainment (Vargo et al., 2021). The city of Charlottesville has a calculated SVI of 0.5727, which is considered medium–high. The surrounding county of Albemarle has an SVI of 0.2469, which is considered low (Flanagan et al., 2011). The U.S. Environmental Protection Agency recently created the Community Health Vulnerability Index (CHVI), which is a tool to help identify vulnerable populations at risk from wildfire smoke exposure, with risk factors including asthma, chronic obstructive pulmonary disease, income, poverty, and unemployment. Based on data from 2008–2018, counties with high CHVI risk had a greater increase in annual number of days with moderate or unhealthy PM_2.5_ levels (Khan et al., 2023). Charlottesville and Albemarle County are categorized in the lowest risk group according to the CHVI.

The impact of infectious disease is an important consideration, notably the ongoing spread of COVID-19, which had summer spikes during this period. In our initial assessment of the raw data, a seasonal effect remained even after removing conditions like influenza and COVID-19, which could reflect the positive impact of higher temperatures on respiratory syndromes (Lin et al., 2009). Despite not considering daily temperature when selecting cases, we believe that controlling for season removes much of any potential confounding. Lastly, the data do not distinguish between the dispositions of patients admitted to the ED.

Although the goal of this paper was to evaluate the impact of the Québec wildfires in summer 2023, there is potential for expansion to include prior episodes in which poor air quality values were linked to fires elsewhere. Alternatively, available PM_2.5_ could be used to assess how much of the typical seasonality we see with respiratory conditions may be attributable to wildfire episodes. Although wildfire occurrences are relatively rare in the eastern USA, they are frequent in the West.

## Conclusion

We examined daily ED visits to the University of Virginia Health System in Charlottesville, VA, from 2017 to 2023. Our goal was to determine if days with elevated PM_2.5,_ arising from the Québec wildfires during the summer of 2023, had an impact on respiratory ED visits. Poor air quality was associated with elevated same day ED departures for respiratory visits and increased odds of visiting the ED for a respiratory complaint. These results were also consistent for males and white individuals, but there was no effect on women or patients from any of our three age-based subgroups.

These results suggest that wildfires and their associated increases in fine particulate matter have a measurable impact on the number and proportion of patients presenting to the ED for respiratory conditions. Prior research has focused mainly on the health impacts for locations and populations near the wildfires. Additional research should be conducted looking at a larger conglomerate of mid-Atlantic EDs to better understand the wildfires’ regional impact, perhaps involving the DC metroplex, Richmond, Virginia, and North Carolina health systems. A larger sample size could further elucidate disproportionate effects on subgroups that other studies have evaluated (Wettstein et al., 2018) and other conditions beyond respiratory complaints. As wildfires increase, the respiratory health of the population may noticeably deteriorate, particularly near and downwind of locations prone to wildfires (such as savannas and Mediterranean climates of the western USA). Furthermore, wildfire smoke may exacerbate illness in patients with chronic conditions, especially those living in areas with generally poor air quality.

As public health officials consider future risks for their populations, it is important to understand that several threats that were not previously part of traditional risk analysis exist now. This includes planning for surges in ED utilization when air quality is poor. This can be particularly challenging given the sparse collection of these data. Future public health data into strategies to reduce exposure, particularly in high-risk populations, through recommendations to stay indoors and improve whole house filtration systems is needed to drive health policy. What can be taken from a public health policy is the recognition that air quality is not fundamentally a local problem but has the potential to be a shared problem.

## Data Availability

Access to the ED data used in this study is governed by the University of Virginia (UVA) Institutional Review Board (IRB) Data Management and Security Plan, which is available from the authors upon request. After the study has ended, anonymized datasets will be made available to other researchers, with the agreement of the authors and the UVA Institutional Review Board, upon request. The research team will decide upon the types of data that may be made available to others, in compliance with the HIPAA laws of the United States of America and UVA IRB policies on data security. The air quality data are readily available from the EPA’s AirNow website.
